# What Should Chinese Medical Students Learn From the COVID-19 Outbreak From Surgeons' View? A Deep Insight Into the Post-COVID-19 Situation

**DOI:** 10.3389/fpubh.2022.736047

**Published:** 2022-03-25

**Authors:** Jun Lu, Dong Wu

**Affiliations:** ^1^Department of Gastric Surgery, Fujian Medical University Union Hospital, Fuzhou, China; ^2^Department of General Surgery, Fujian Medical University Union Hospital, Fuzhou, China; ^3^Key Laboratory of Ministry of Education of Gastrointestinal Cancer, Fujian Medical University, Fuzhou, China

**Keywords:** medical education, COVID-19, pandemic, Chinese, learning

An outbreak of novel coronavirus (COVID-19) emerged in late December 2019 in Wuhan city, China and spread quickly. Given the seriousness of the disease, this event was declared a public health emergency of international concern (PHEIC) on Jan 30, 2020 by WHO. The COVID-19 knows neither politics nor borders (1, 2). As of 30 January 2022, over 370 million confirmed cases and over 5.6 million deaths have been reported globally (3). According to the summary of a report of 72,314 cases from China, the overall case-fatality rate was 2.3% (4). Hopefully, it is true that COVID-19 has a lower lethality rate than SARS.

In response to COVID-19, surgeons have to rethink almost every aspect of their daily clinical practice. Elective surgery has been canceled and clinic services are scaled back regardless of the health system or geographical boundaries. However, what should medical students learn from this outbreak? First of all, strive to be a human with a strong sense of social responsibility and mission. Surgical education aims to provide students with a foundation of surgical knowledge, practice, and technique. Before that, however, surgical trainees were primarily medical students, who must stand side-by-side with all members of the health care teams to respond to changes in clinical needs caused by the COVID-19 pandemic. According to CDC of China, the extraordinary containment efforts on the Chinese frontline have come at a high price—over 3,000 Chinese medical professionals have been infected with COVID-19 and at least 10 have lost their lives, including surgeons (5). They are the heroes of humanity that should never be forgotten, even though they are only fathers, mothers, sons, or daughters at home. When the next outbreak comes, our medical students will be as responsible as their predecessors. Secondly, strive to be a medical professional with a humanitarian and compassionate outlook. Since the outbreak, tens of thousands of medical workers have devoted themselves to the intensive work of treating patients (6). Whichever frontline they are fighting on, health workers everywhere follow the same oath to treat the sick. Actually, the State Council of China introduced bold plans to revolutionize surgical education on July 11, 2017 (7). Professional attitudes such as compassion, empathy, and ethics must become as recognizable a mark of surgical doctors or medical students in China as a stethoscope. Third, commitment to basic or epidemiology research should be encouraged, even for surgical graduate students and postdocs. This will encourage more medical students to engage in basic medical research and epidemiological research. It has been proved by the COVID-19 outbreak that medical and health research saves countless lives. Although valuable lessons were learned from the SARS epidemic (8), larger systemic changes are still needed so that we can respond more efficiently and effectively when the next epidemic arrives. Fourth, our students need to be able to release their mental stress properly. For a prolonged period since the beginning of the outbreak, the government encouraged people to stay at home; postponed or canceled large public events; and closed schools and universities. This has made many students feel uneasy, especially graduate students of the graduating classes, because they usually have a lot of research to complete by the month of May. West China Hospital, one of the top tertiary hospitals in China has come to a halt; its graduate students and interns are prohibited from returning to school and work. In addition, this hospital has set up special psychological intervention counseling services through telephone and Internet platforms to help people in need, and published a psychological protection manual for free download (9). This is similar to the measures taken by the author's university. Last but not the least, our surgical students should learn to use social media correctly during such community-wide quarantine. In the author's team, the supervisors often hold webcam meetings for medical students to steadily facilitate learning progress. This is the epitome of China's countless university hospitals today. Every coin has two sides. Too much information, not balanced or filtered, may lead to poor healthcare decisions (10). Even worse, the social media panic and misinformation traveled faster than the coronavirus itself (11). Our medical students, as the important bridge between policy makers and the public, should be wise about social media information.

Another point that needs to be highlighted is the violence against medical staff or health professionals, which was a major factor that made several high caliber students second guess their intention to become surgeons. Although it affects health workers in nearly all healthcare settings, the scale, frequency, and viciousness of attacks on surgeons in China are particularly severe (12). It is infuriating that even in the midst of the fight against COVID-19, surgical staff members were violently hurt by our patients. Therefore, it is our sincere hope that health-care professionals will be respected and protected from any violence.

With COVID lasting, the emergence of the new variants, such as alpha, beta, delta, and Omicron SARS-CoV-2 variants, were associated with new waves of infections, sometimes across the entire world (13, 14). In the future, COVID may co-exist with human for a long time just like other virus. Above mentioned measures and viewpoints may still provide effective guidance for medical students, especially for those majored in surgery.

Likewise, similar opinions also apply to other medical, dental or nursing specialties (15–19). It is believed that people and medical personnel all over the world can work together to overcome the battle without smoke. We expect Chinese medical students to grow better after this outbreak. We are also grateful that Western surgical scientists are concerned about surgical education in China.

## Author Contributions

JL and DW: conception, design, collection, assembly of data, data interpretation, and manuscript writing. Both authors read and approved the final manuscript.

## Conflict of Interest

The authors declare that the research was conducted in the absence of any commercial or financial relationships that could be construed as a potential conflict of interest.

## Publisher's Note

All claims expressed in this article are solely those of the authors and do not necessarily represent those of their affiliated organizations, or those of the publisher, the editors and the reviewers. Any product that may be evaluated in this article, or claim that may be made by its manufacturer, is not guaranteed or endorsed by the publisher.
